# Neutron detection performance of gallium nitride based semiconductors

**DOI:** 10.1038/s41598-019-53664-7

**Published:** 2019-11-26

**Authors:** Chuanle Zhou, Andrew G. Melton, Eric Burgett, Nolan Hertel, Ian T. Ferguson

**Affiliations:** 1 Department of Electrical and Computer Engineering, Missouri University of Science and Technology, Missouri, 65409 USA; 2 Department of Electrical and Computer Engineering, University of North Carolina at Charlotte, Charlotte, North Carolina, 28223 USA; 3 Nuclear Engineering Program, Idaho State University, Pocatello, Idaho 83209 USA; 4 Nuclear Engineering Program, Georgia Institute of Technology, Atlanta, Georgia 30332 USA; 5 Southern Polytechnic College of Engineering and Engineering Technology, Kennesaw State University, Marietta, GA 30060 USA

**Keywords:** Electrical and electronic engineering, Optical sensors

## Abstract

Neutron detection is crucial for particle physics experiments, nuclear power, space and international security. Solid state neutron detectors are of great interest due to their superior mechanical robustness, smaller size and lower voltage operation compared to gas detectors. Gallium nitride (GaN), a mature wide bandgap optoelectronic and electronic semiconductor, is attracting research interest for neutron detection due to its radiation hardness and thermal stability. This work investigated thermal neutron scintillation detectors composed of GaN thin films with and without conversion layers or rare-earth doping. Intrinsic GaN-based neutron scintillators are demonstrated via the intrinsic ^14^N(n, p) reaction, which has a small thermal neutron cross-section at low neutron energies, but is comparable to other reactions at high neutron energies (>1 MeV). Gamma discrimination is shown to be possible with pulse-height in intrinsic GaN-based scintillation detectors. Additionally, GaN-based scintillation detector with a ^6^LiF neutron conversion layer and Gd-doped GaN detector are compared with intrinsic GaN detectors. These results indicate GaN scintillator is a suitable candidate neutron detector in high-flux applications.

## Introduction

Radiation detectors, including the detection of α-particles, X-ray, electrons, and neutrons, are important tools in the areas of particle physics, nuclear power, and international security. The most commonly used neutron detection material is ^3^He, which is an extremely rare and expensive isotope. The global shortage of ^3^He has generated tremendous interest in alternative neutron-sensitive materials for detection, and it is desirable to transition to solid-state neutron detectors as these offer superior mechanical robustness and lower voltage operation compared to gas detectors.

Scintillators are another type of radiation detector which absorb neutrons and emit relatively low energy (between ultraviolet to visual) photons, which can then be detected by a photodetector such as a photomultiplier tube (PMT) or avalanche photodiode (APD). Silicon^1,2^ (Si) and Gallium Arsenide^3,4^ (GaAs) based detectors have been investigated due to their relatively low cost and wide availability. Wide bandgap materials, such as SiC, diamond and ZnO, are of great interest for radiation detection due to their low radiation damage and high temperature stability^5–7^.

Gallium Nitride (GaN) is an attractive alternative material due to its superior radiation hardness^8,9^, thermal stability, and its status as both a mature optoelectronic material^10^ and an emerging material for electronic applications. GaN-based devices have been investigated for α-particle and X-ray detection, showing promising results over large temperature range, and under high radiation dose^11^. GaN-based Schottky diode detectors have been recently explored for neutron detection with ^6^Li, ^10^B, or ^157^Gd conversion layers or rare-earth doping^12,13^, with good radiation resist level up to 10^16^ n/cm^2^ neutron fluences^14^.

**Figure 1:**
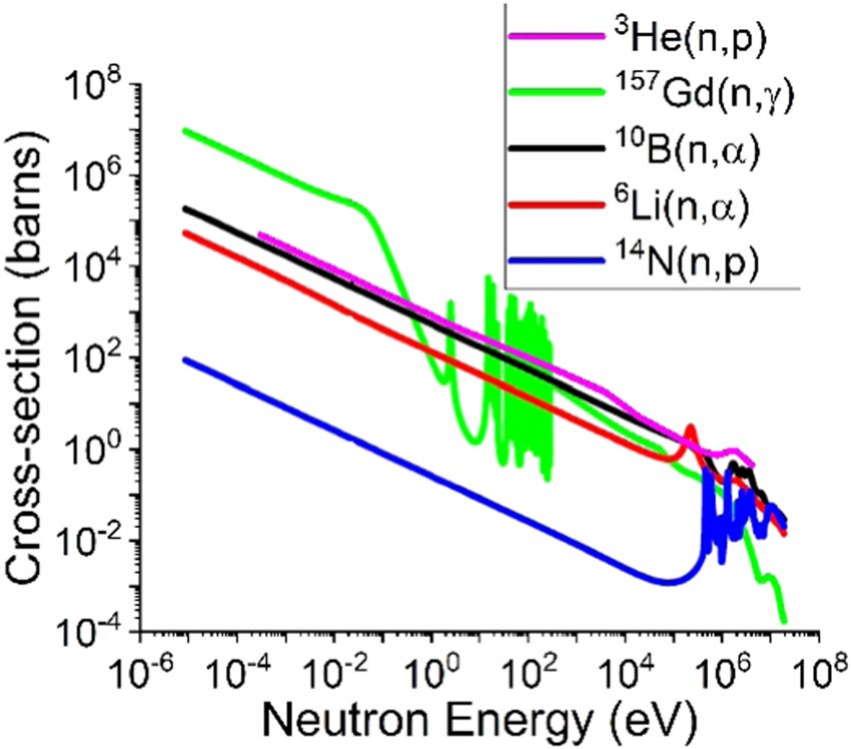
Cross-section for neutron reactions of interest.

This work reports GaN for intrinsic neutron detection through the ^14^N(n, p) reaction, which could replace the expensive conversion layers. A polycrystalline Aluminum Nitride (AlN) resistive neutron detector utilizing the ^14^N(n, p) reaction has been previously reported and was shown to scale linearly with reactor power^15^. There are few studies for GaN-based intrinsic neutron detectors^16^, typically due to the small cross-section for ^14^N(n, p) thermal neutron reaction, Fig. 1. However, the intrinsic GaN neutron scintillator is of great interest due to the following factors. First, nitrogen-14 (^14^N) accounts for 99.6% of naturally occurring nitrogen isotopes, so there is no need for isotope enrichment, unlike detectors utilizing ^6^Li or ^10^B. Secondly, ^14^N makes up to 50% of the GaN crystal structure, so the density of active nuclei is 4.4 × 10^22^ cm^−3^. Thirdly, ^14^N(n, p) reaction cross-section is comparable to other reactions at high neutron energies (>1 MeV), Fig. 1^17^. In the ^14^N(n, p) reaction, the ^14^N nucleus absorbs a neutron and emits a proton, thus being transmuted to ^14^C, Eq. (1).

1 $${}_{7}{}^{14}{\rm{N}}{+}_{0}^{1}{\rm{n}}\to {}_{6}{}^{14}{\rm{C}}+{}_{1}{}^{1}{\rm{p}}$$

The total Q-value of the ^14^N(n, p) reaction is 625.7 keV, with the ejected proton receiving 584 keV. A major advantage of GaN-based materials is the ability to integrate any potential neutron response into pre-existing device technology, such as photodetectors.

In this work, un-doped and silicon (Si) doped GaN are fabricated and demonstrated as neutron scintillator via the ^14^N(n, p) reaction. Gamma discrimination between gamma rays and neutrons is calibrated. The GaN detectors with LiF conversion layer or doped with Gd are also characterized and compared with intrinsic GaN scintillator.

## Experimental Set Up

The epitaxial GaN scintillators were grown in a highly modified EMCORE D-125 rotating disk metal organic chemical vapor deposition (MOCVD) reactor with a short jar configuration. Initially, a 2 μm thick, unintentionally-doped GaN template layer was deposited on *c*-plane (0001) double-side polished sapphire substrates, employing a two-step heteroepitaxy process consisting of an approximately 20 nm thick low-temperature nucleation/buffer layer followed by a high-temperature layer grown at 1050 °C using trimethylgallium (Ga(CH_3_)_3_) and ammonia (NH_3_) as precursors. This work focused on one 10 µm thick unintentionally doped GaN film, one 10 µm thick Si-doped GaN film, and one Gd-doped GaN. Silane (SiH_4_) was the precursor used for Si-doping. Two different metalorganic precursors for Gd were used in this work: tris(cyclopentadienyl)gadolinium (Cp_3_Gd) and tris(2,2,6,6-tetramethyl-3,5-heptanedionato)gadolinium (Gd(TMHD)_3_). The growth process and epitaxial layer thickness were monitored *in-situ* using an optical reflectometer.

The crystalline quality of the GaN layers was analyzed by high-resolution X-ray diffraction (HR-XRD) omega scans. Luminescence and self-absorption characteristics were studied using front and back collected photoluminescence (PL) at room temperature (300 K) with a helium-cadmium (He-Cd) laser excitation source with wavelength λ = 325 nm. In the back-collected configuration, luminescence was collected after passing through the sample and the sapphire substrate.

Scintillation was detected by coupling the GaN films to a PMT with enhanced ultraviolet sensitivity using a thermal neutron source of AGN-201 homogeneous thermal reactor. Scintillators were compared for undoped, Si-doped, and Gd-doped with and without a 5 μm thick enriched ^6^LiF neutron conversion layer, which absorbs neutrons and emits 2.05 MeV alpha particles. Scintillation pulses were counted using a pulse-shaping amplifier and multichannel analyzer (MCA) which includes Hamamatsu R2059 photomultiplier tube and Ortec NIM electronics.

## Results and Discusstion

### III.a Structural characterization results

**Figure 2:**
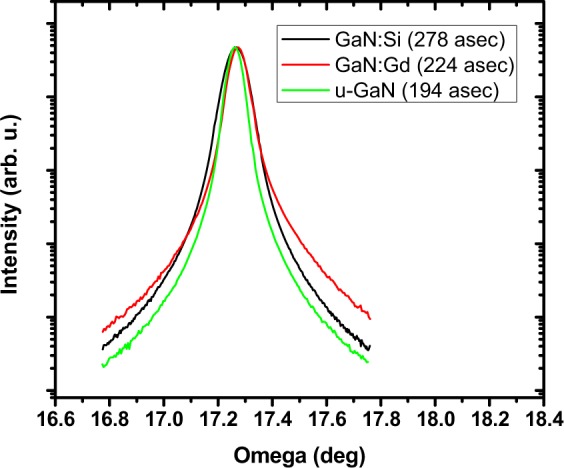
Omega rocking curves of GaN scintillators.

HR-XRD omega scans of the 002 reflection were performed for both the unintentionally doped, Si-doped and Gd-doped GaN films which had full width at half maximum (FWHM) of 194 arcsec, 278 arcsec, and 224 arcsec, respectively, indicating all these samples had very good crystal quality, Fig. 2. Contactless resistivity mapping was used to estimate the free electron concentration in both samples, which was approximately 1 × 10^16^ cm^−3^ for the unintentionally-doped film and approximately 5 × 10^18^ cm^−3^ for the Si-doped film.

**Figure 3:**
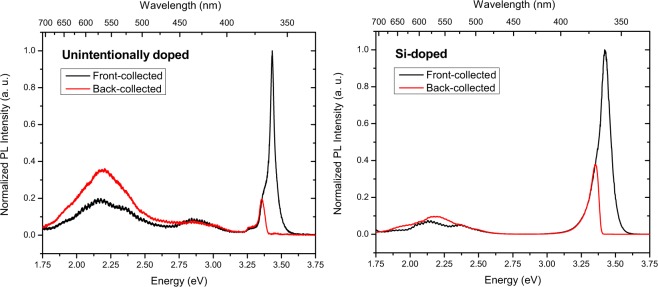
Front- and back-collected PL spectra for unintentionally doped and Si-doped GaN scintillators, illustrating self-absorption of near-band-edge luminescence and enhancement of yellow luminescence.

PL spectra showed that near-band-edge emission in the Si-doped film is approximately an order of magnitude more intense than in the unintentionally doped film, which is typical for these films, indicating that Si-doping created few additional optical traps^18^. The near-band-edge emission peak in the Si-doped film, as expected, is also red-shifted (from 3.433 eV to 3.421 eV) and broadened (FWHM from 0.038 eV to 0.092 eV) with respect to the unintentionally doped GaN. The back-collected PL spectra showed significant self-absorption of near-band-edge emission in both films. Figure 3 shows front- and back-collected PL spectra for both samples. The back-collected spectra have been normalized by ratio of the back-collected peak intensity to the front-collected intensity at the same energy and corrected for any scattering at the sapphire substrate.

The defect-related yellow luminescence, typically centered at 2.2 eV, is observed in both the unintentionally doped and Si-doped, while blue luminescence centered at approximately 2.9 eV is visible only in the unintentionally doped film. Yellow luminescence appears to be enhanced in both back-collected spectra compared to front-collected spectra. There are two contributions to this; optical pumping of defect states by near-band-edge luminescence and minimal re-absorption of sub band-gap photons. In the case of neutron-induced scintillation, theoretical calculations show that the neutrons are absorbed approximately evenly throughout the complete thickness of the film. Thus, scintillation photons will be generated uniformly throughout and, as a consequence, be subject to varying degrees of self-absorption before exiting the film.

### III.b Neutron Detection Results

**Figure 4:**
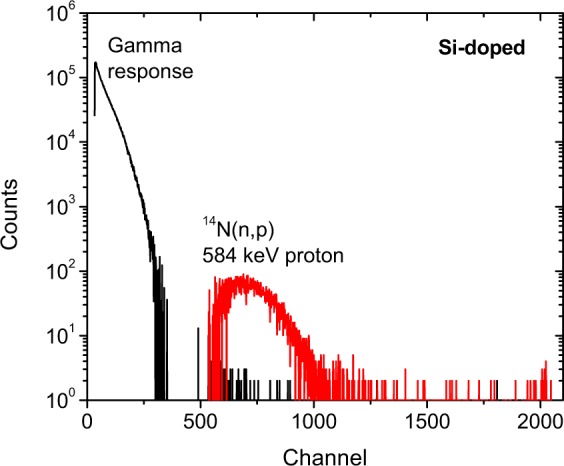
Gamma and thermal neutron scintillation spectra produced by bare Si-doped GaN scintillator.

Pulse height spectra produced by the unintentionally doped and Si-doped GaN scintillators were measured when exposed to reactor gamma rays and thermal neutrons. Reference spectra were also measured with a >1 mm thick cadmium (Cd) shield in place, which effectively absorbs all thermal neutrons. The thermal neutron-response of each scintillator was isolated by subtracting the reference spectrum from the unshielded spectrum. The reference spectrum (which is simply the gamma response of the material) and the neutron-induced spectrum are plotted in Fig. 4. The neutron-induced peak is attributed to ionization from 584 keV protons produced by the ^14^N(n, p) reaction. This is because it is the only neutron reaction in GaN that produces a heavy charged particle, as gallium interacts with neutrons primarily through elastic scattering. Broadening of the neutron-induced peak is caused by the distribution of photon-generation events in the GaN film. The gap between the gamma- and neutron- induced peaks in the Si-doped GaN film shown in Fig. 4 indicates the possibility for effective “gamma discrimination” based on pulse height, which is critical for neutron detection. The ratio of neutron-induced events to gamma-induced events is 78:1 for a cutoff at channel 500. The neutron-induced scintillation efficiency for 10 µm thick Si-doped GaN was one count per 2.55 × 10^7^ neutrons, which is lower than the theoretical efficiency of one count per 1.27 × 10^4^ neutrons. This is likely due to optical losses from self-absorption and total internal reflection in the GaN film; however, this data clearly shows that GaN is sufficient for neutron detection but could still be further optimized.

**Figure 5:**
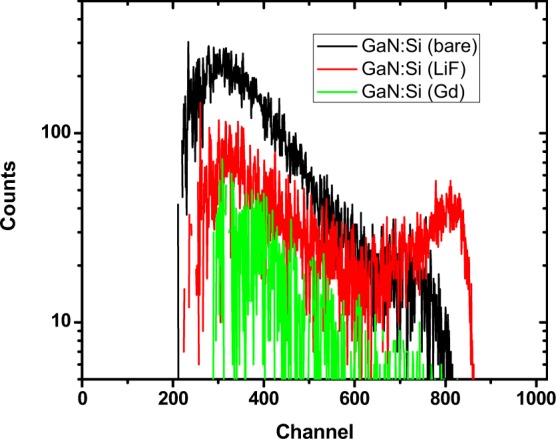
Neutron-induced luminescence in Si-doped GaN with varying conversion layers.

Alpha particle response for 10 μm thick undoped, Si-doped, and Gd-doped scintillators are shown in Fig. 5^12^. The Si-doped GaN scintillator showed a distinct alpha-induced peak with very good gamma-discrimination, while the undoped and Gd-doped scintillators did not show a distinct alpha peak. This is due to the lower optical efficiency of these films relative to the Si-doped film, as seen in PL measurements. A material that produces fewer photons produced for a given alpha ionization trail will show these light pulses at lower channel numbers. In the undoped and Gd-doped films the alpha-induced events were compressed to the point that they occupied the same channel numbers as gamma-induced pulses, and thus gamma discrimination was not possible^12^.

**Figure 6:**
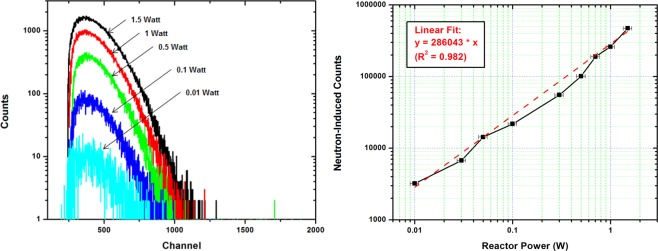
(**a**) Neutron-induced scintillation spectra produced by ^6^LiF-coated Si-doped GaN scintillator. (**b**) Integrated neutron count dependence on reactor power with linear fit (dashed line). The AGN-201 reactor has a power-dependent flux of 5.3 × 10^6^ thermal neutrons per cm^2^ per second per Watt.

The Si-doped GaN scintillator was also tested with an enriched ^6^LiF neutron conversion layer, which absorbs thermal neutrons and emits 2.05 MeV alpha particles. Distinct peaks caused by the 2.05 MeV alpha particle were observed in neutron-induced scintillation spectra. Figure 6 shows the results of a reactor linearity test in which scintillation spectra were recorded from the Si-doped GaN scintillator with the conversion layer at a range of reactor power levels. Scintillation spectra were collected for 300 seconds. Figure 6(b) shows the integrated neutron-induced counts from the detector plotted versus reactor power with a linear fit having a coefficient of determination (R^2^) of 0.982, which indicates that the scintillator detector is linear over approximately three orders of magnitude of reactor power. Based on the linear fit, the ^6^LiF-coated GaN scintillator produces 286,043 neutron-induced pulses per Watt of reactor power. This equates to a detector efficiency of one count per 1.1 × 10^5^ neutrons, which is lower than the maximum theoretical efficiency of one count per 3.5 × 10^1^ neutrons (based on the macroscopic cross-section of the ^6^LiF conversion layer). The observed efficiency of the ^6^LiF coated detector was two orders of magnitude better than the bare Si-doped GaN detector. The same optical inefficiencies (self-absorption and total internal reflection) attenuate the scintillation in the ^6^LiF-coated detector. However, additional deviation from the theoretical neutron absorption efficiency arises from partial energy deposition from the secondary alpha and triton particles in the conversion layer prior to entering the GaN scintillator. This source of inefficiency is unavoidable in conversion layer detectors.

## Conclusion

This work examined thermal neutron-sensitive scintillators composed of epitaxial GaN thin films. The PL spectra of the scintillators showed that the Si-doped GaN scintillator had higher luminescent efficiency and less self-absorption than the unintentionally doped scintillator. Neutron-induced scintillation was observed in the Si-doped film, which is of the first reported observations of the ^14^N(n, p) reaction in GaN. This result shows that GaN is an intrinsically neutron-sensitive material that can be used for neutron detection. Furthermore, the Si-doped GaN scintillation spectra showed excellent gamma-discrimination based on pulse height, which is a key characteristic of neutron detectors, while the undoped and Gd-doped scintillators did not show gamma discrimination. Finally, a ^6^LiF-coated GaN scintillator was tested and exhibited a linear response to reactor power. The radiation hardness of GaN and the demonstrated linear response to reactor power make these scintillators potentially suitable for high flux applications, such as nuclear reactor power monitoring.

## References

[1] Conway AM, Wang TF, Deo N, Cheung CL, Nikolic RJ (2009). Numerical Simulations of Pillar Structured Solid State Thermal Neutron Detector: Efficiency and Gamma Discrimination. IEEE Trans. Nucl. Sci..

[2] Bellinger SL, McNeil WJ, Unruh TC, McGregor DS (2009). Characteristics of 3D Micro-Structured Semiconductor High Efficiency Neutron Detectors. IEEE Trans. Nucl. Sci..

[3] McGregor DS, Klann RT, Gersch HK, Yang YH (2001). Thin-film-coated bulk GaAs detectors for thermal and fast neutron measurements. Nucl. Inst. Meth. Phys. Res. A.

[4] Thompson AV, Mares JW, Seigneur H, Schoenfeld WV (2007). Optimization of GaAs PIN diodes for neutron detection. Proc. SPIE.

[5] Sellin PJ, Vaitkus J (2006). New materials for radiation hard semiconductor dectectors. Nucl. Instrum. Methods A.

[6] Kumar S, Reshi BA, Varma R (2018). Comparison of Silicon, Germanium, Gallium Nitride, and Diamond for using as a detector material in experimental high energy physics. Results Phys..

[7] J. E. Nause, E. A. Burgett, N. E. Hertel and I. Ferguson Thin film doped ZnO neutron detector, US patent US20130075718A1 (2012).

[8] Polyakov AY (2013). Radiation effects in GaN materials and devices. J. Mater. Chem. C.

[9] Ionascut-Nedelcescu (2002). Radiation hardness of gallium nitride. IEEE Trans. Nucl. Sci.,.

[10] Li T (1999). Improved ultraviolet quantum efficiency using a semitransparent recessed window AlGaN/GaN heterojunction p-i-n photodiode. Appl. Phys. Lett..

[11] Zhu Z (2018). High-temperature performance of gallium-nitride-based pin alpha-particle detectors grown on sapphire substrates, *Nuclear Inst. and Methods in Physics*. Research, A.

[12] Melton, A. *et al*. GaN as a Neutron Detection Material, *Proceedings of the IEEE Southeast Con*, **11261428** (2010).

[13] Melton AG, Burgett E, Xu T, Hertel N, Ferguson IT (2012). Comparison of neutron conversion layers for GaN-based scintillators. Phys. Status solidi C.

[14] Mulligan P, Wang J, Cao L (2013). Evaluation of freestanding GaN as an alpha and neutron detector, *Nuclear Inst. and Methods in Physics*. Research A.

[15] Moon BS, Yoo DS, Hwang IK, Chung CE, Holcomb DE (2007). Flux measurements in a nuclear research reactor by using an aluminum nitride detector. Nucl. Instr. Meth. Phys. Res. B.

[16] Wang J, Mulligan P, Brillson L, Cao LR (2015). Review of using gallium nitride for ionizing radiation detection. Appl. Phys. Rev..

[17] National Nuclear Data Center. Available: http://www.nndc.bnl.gov.

[18] Ramvall P (2000). Doping-dependent optical gain in GaN. Appl. Phys. Lett..

